# Supplementary material for: Synthesis and evaluation of radiogallium-labeled long-chain fatty acid derivatives as myocardial metabolic imaging agents
Source: PLoS One. 2021 Dec 15;16(12):e0261226. doi: 10.1371/journal.pone.0261226 (PMC8673672; doi:10.1371/journal.pone.0261226)
Supplement: S5 Fig — (PDF) [file pone.0261226.s005.pdf]

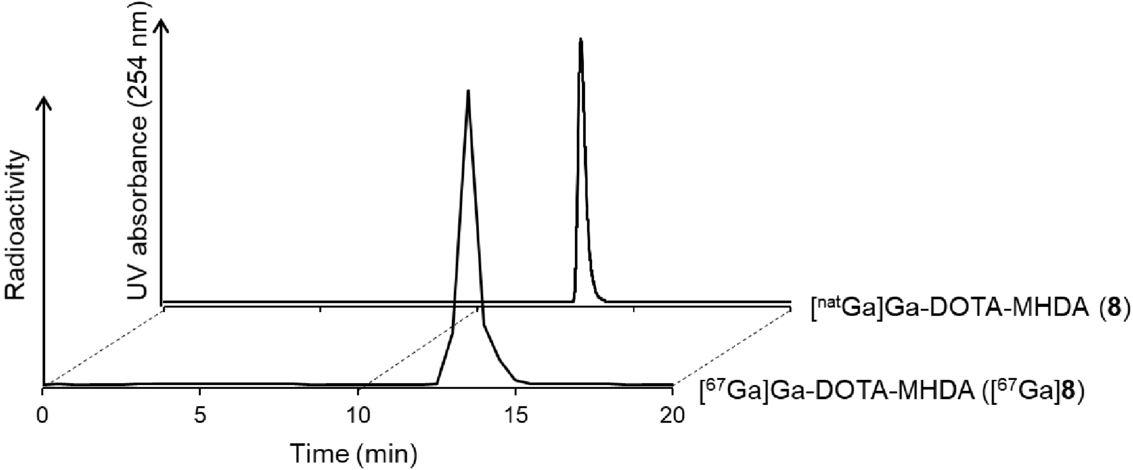

Fig S8. HPLC chromatograms of [^{nat}Ga]Ga-DOTA-MHDA (**8**) and [^{67}Ga]Ga-DOTA-MHDA ([^{67}Ga]**8**). HPLC system: Cosmosil 5C$_{18}$-AR-II column (4.6 mm ID × 150 mm) at a flow rate of 1.0 mL/min with a gradient mobile phase of 70–95% methanol in water with 0.1% TFA for 20 min, with UV detector at 254 nm wavelength, column temperature: 40 °C.
