# Supplementary material for: Synthesis and evaluation of radiogallium-labeled long-chain fatty acid derivatives as myocardial metabolic imaging agents
Source: PLoS One. 2021 Dec 15;16(12):e0261226. doi: 10.1371/journal.pone.0261226 (PMC8673672; doi:10.1371/journal.pone.0261226)

^{1}H-NMR: **1** (DMSO-*d*$_6$)

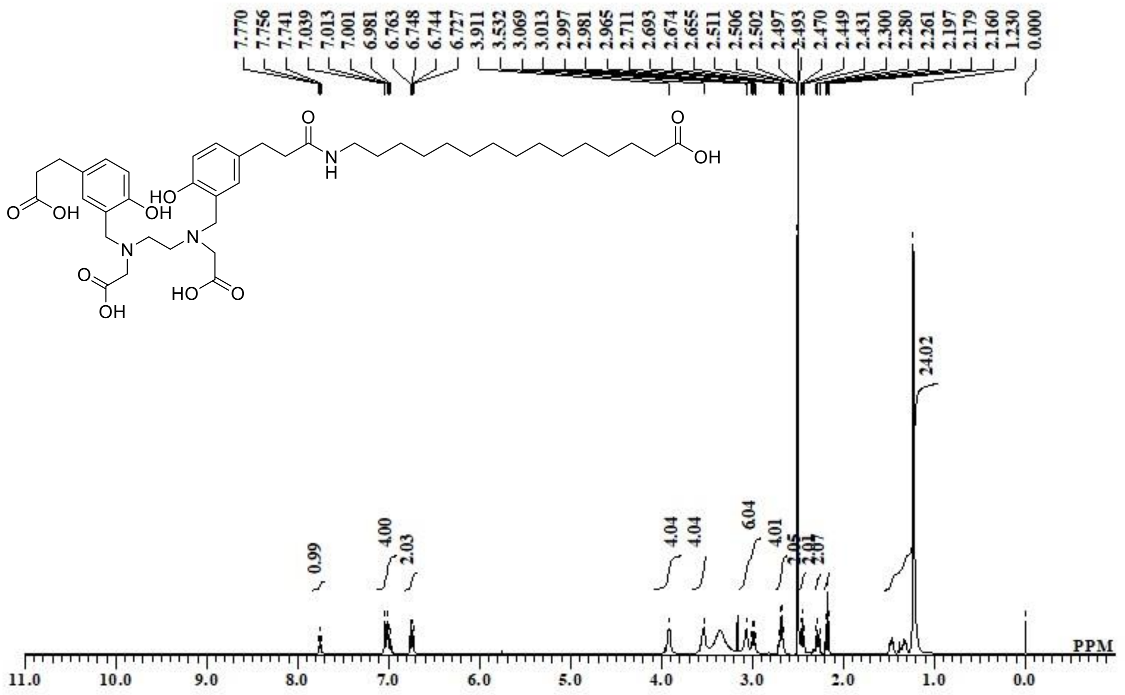

^{1}H-NMR: **2** (DMSO-*d*$_6$)

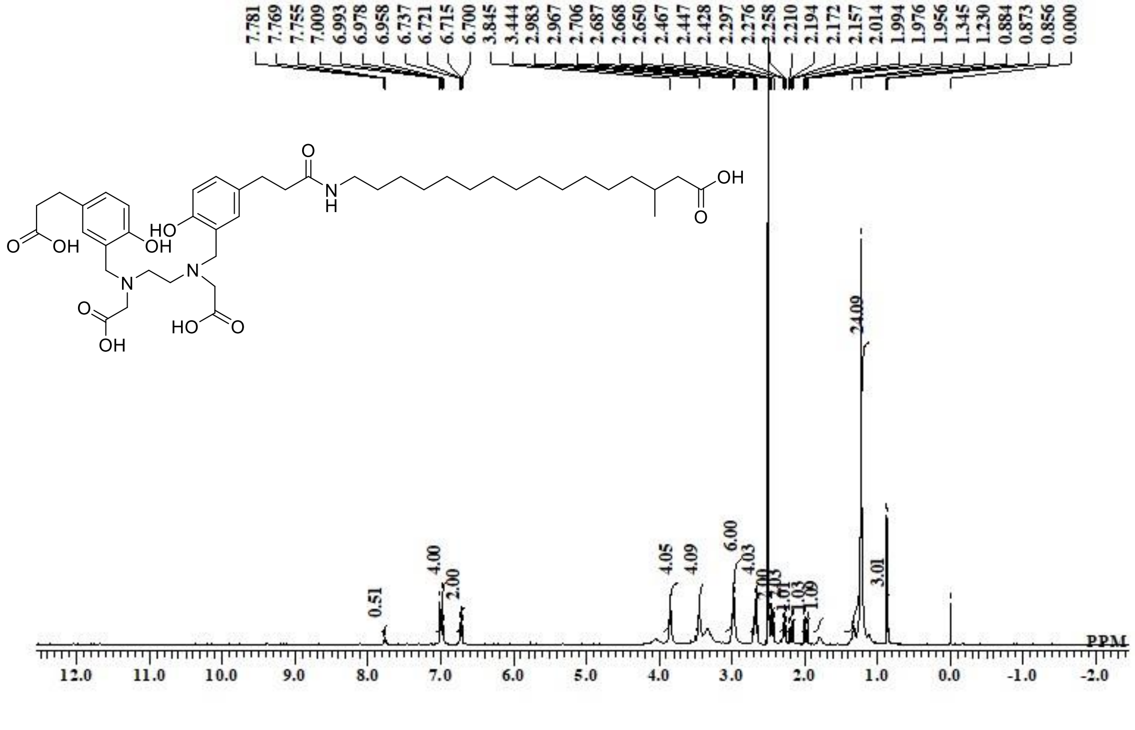

^{1}H-NMR: **3** (CD_3OD)

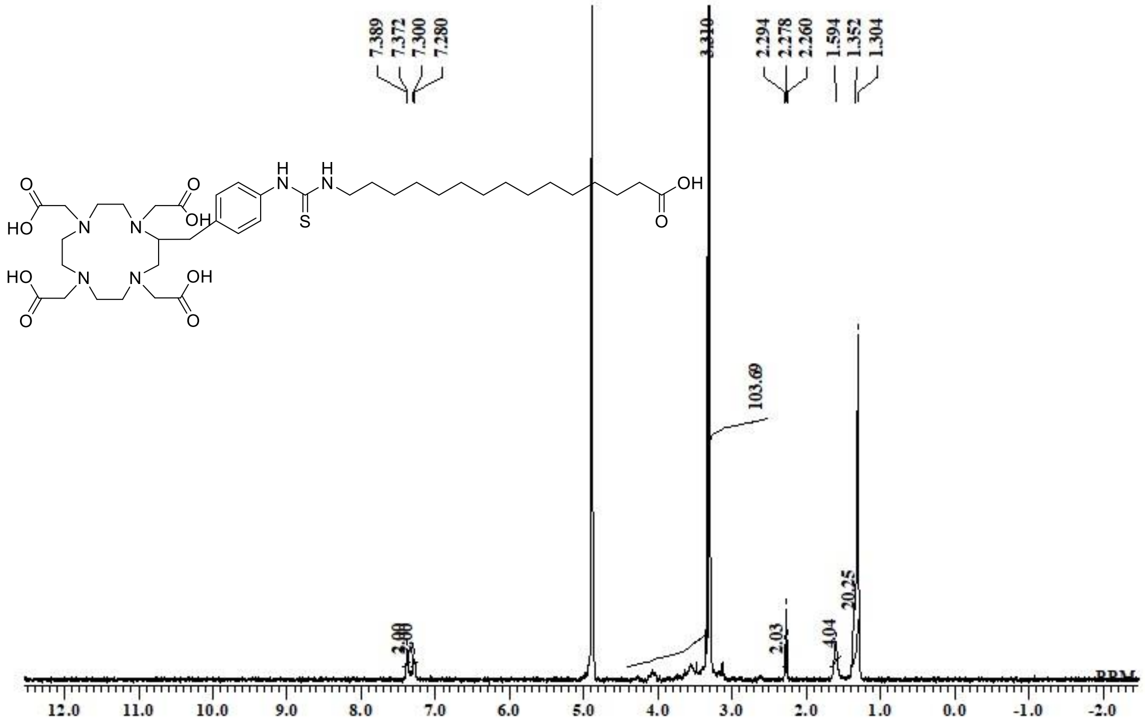

^{1}H-NMR: **4** (CD_3OD)

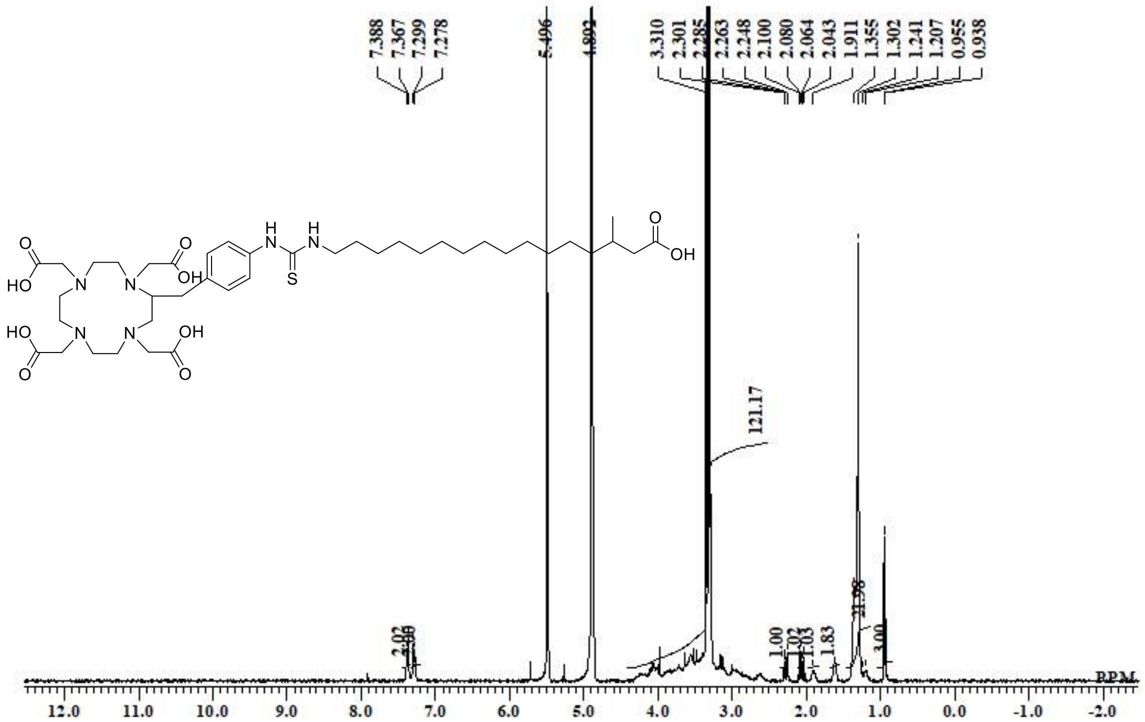

^{1}H-NMR: **10** ($CDCl_3$)

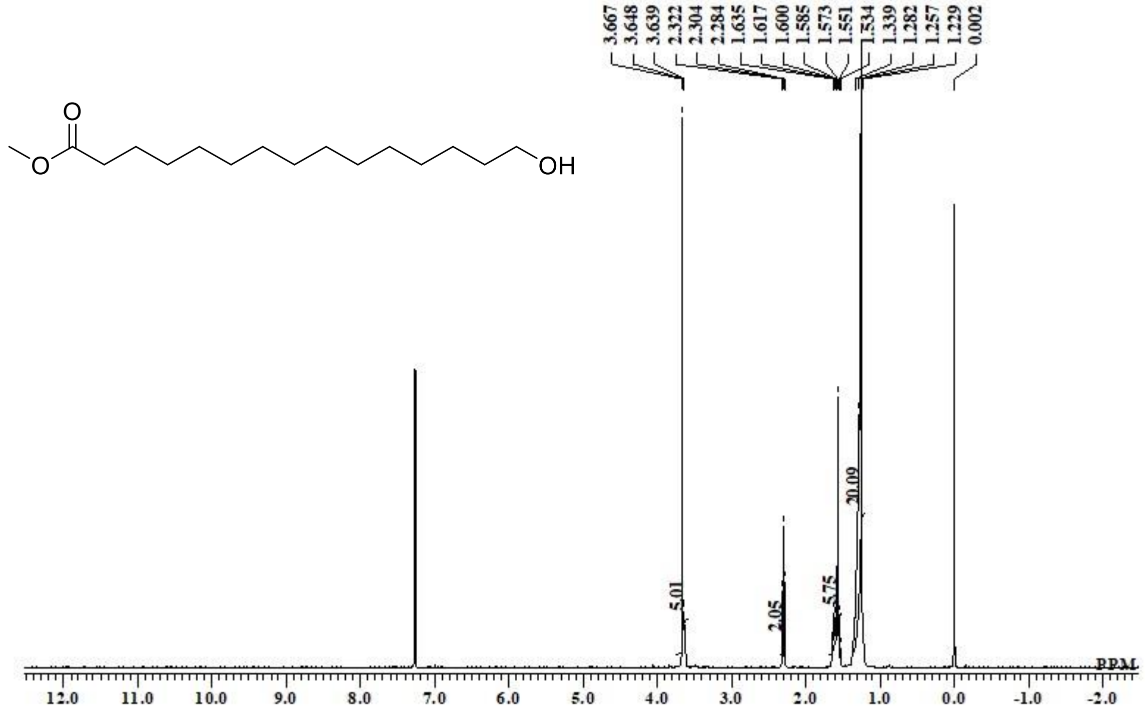

^{1}H-NMR: **11** ($CDCl_3$)

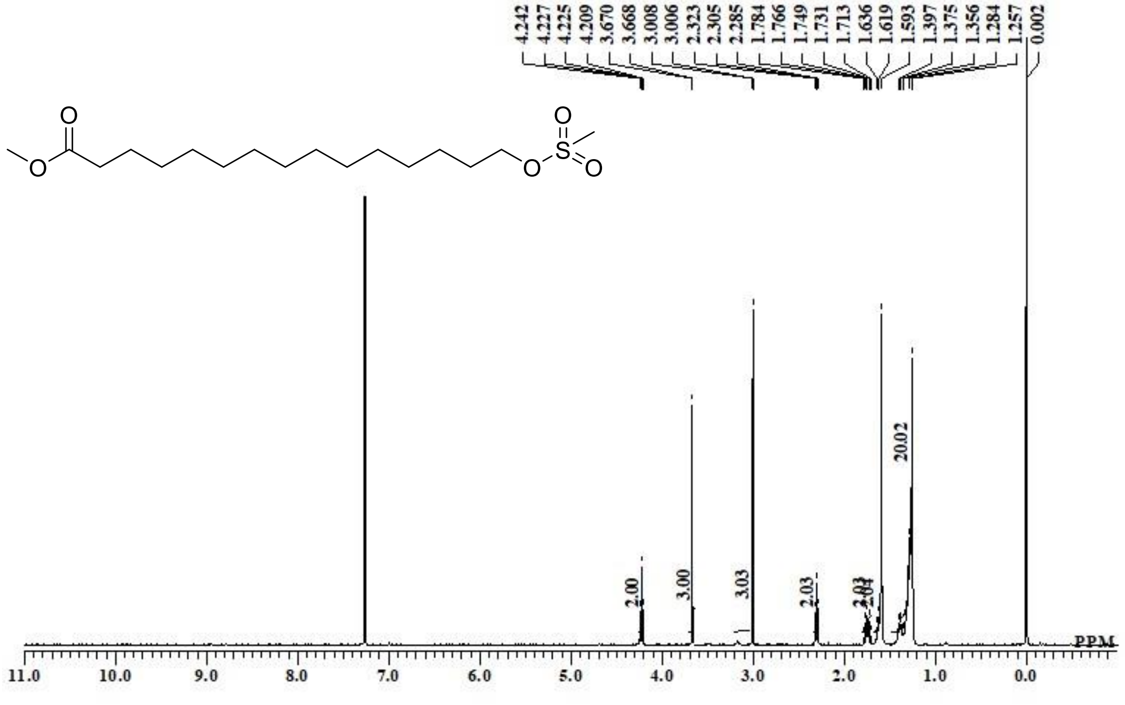

^{1}H-NMR: **12** ($CDCl_3$)

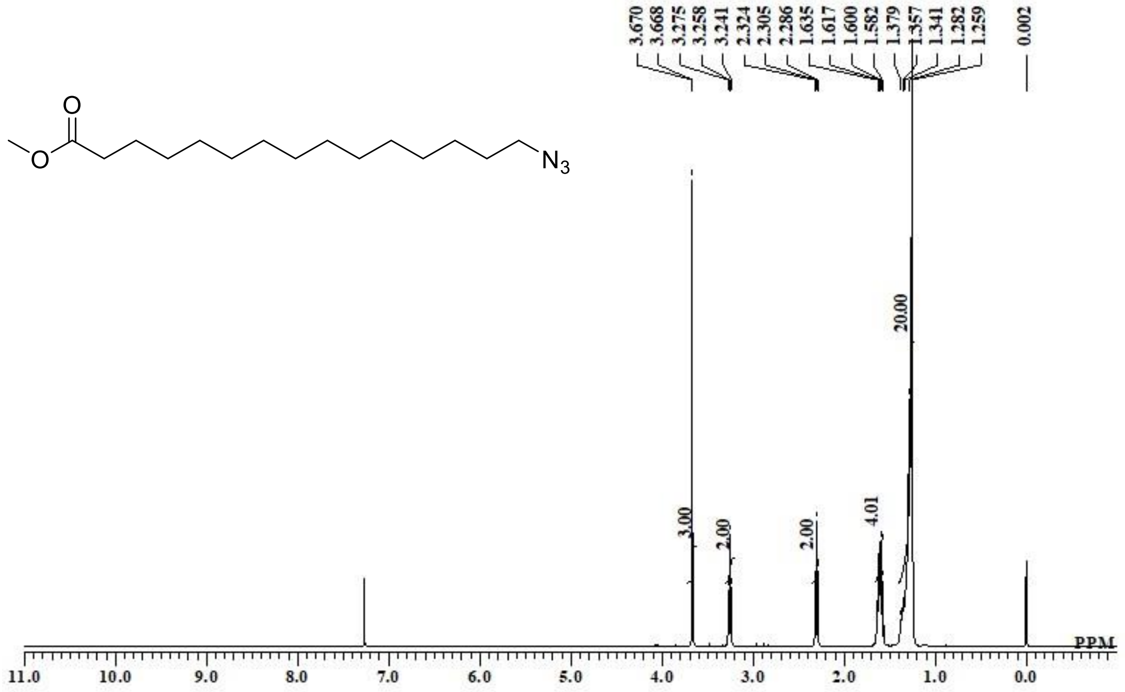

^{1}H-NMR: **13** ($CDCl_3$)

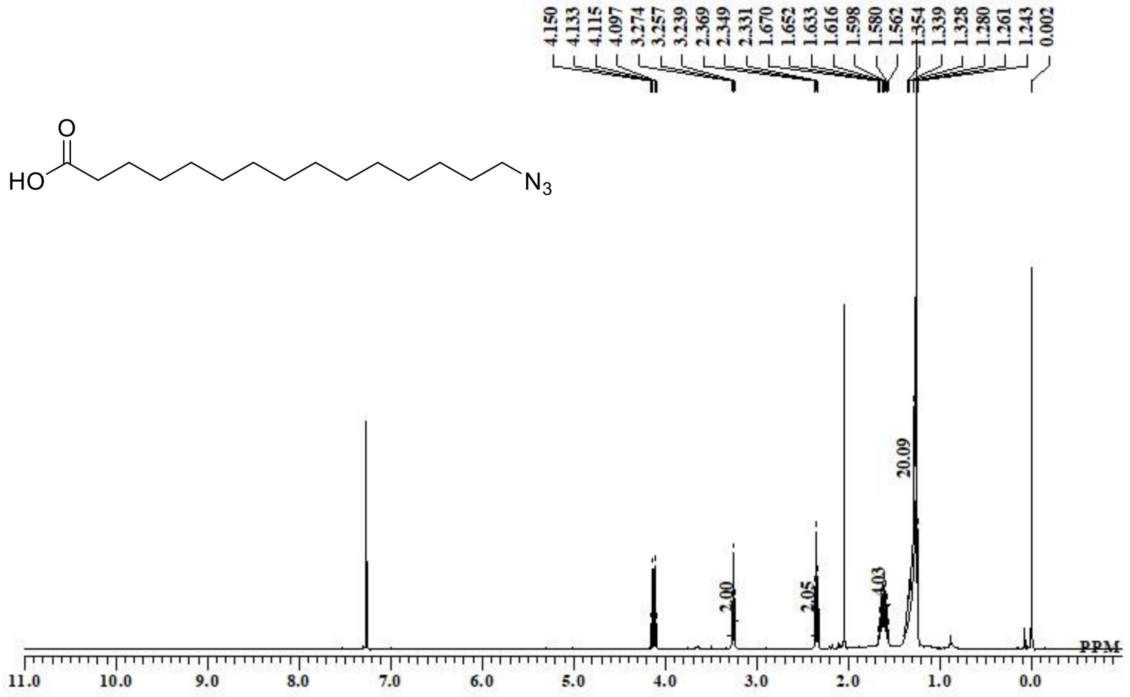

^{1}H-NMR: **14** (CD_3OD)

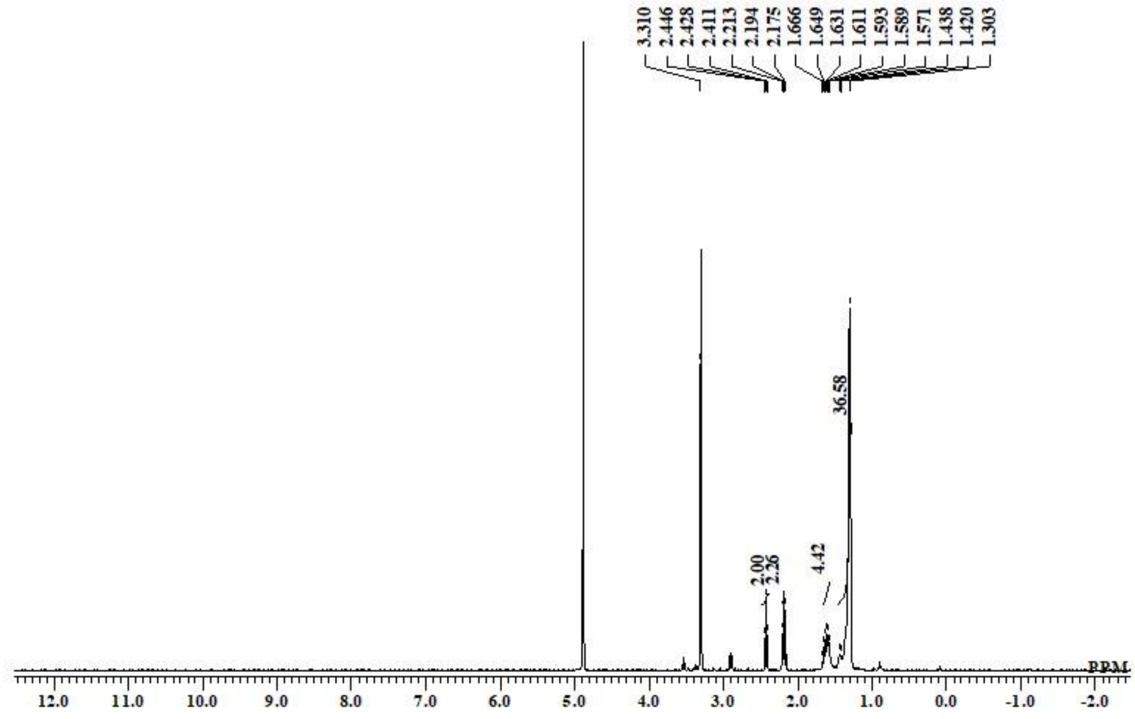

^{1}H-NMR: **16** ($CDCl_3$)

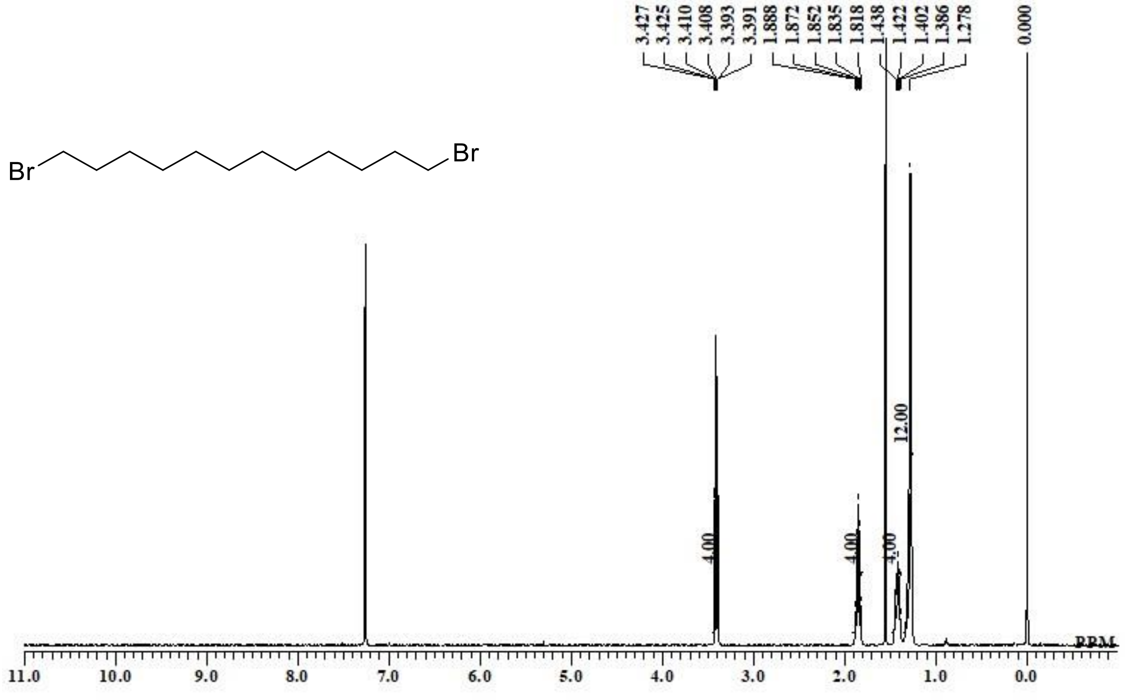

^{1}H-NMR: **17** ($CDCl_3$)

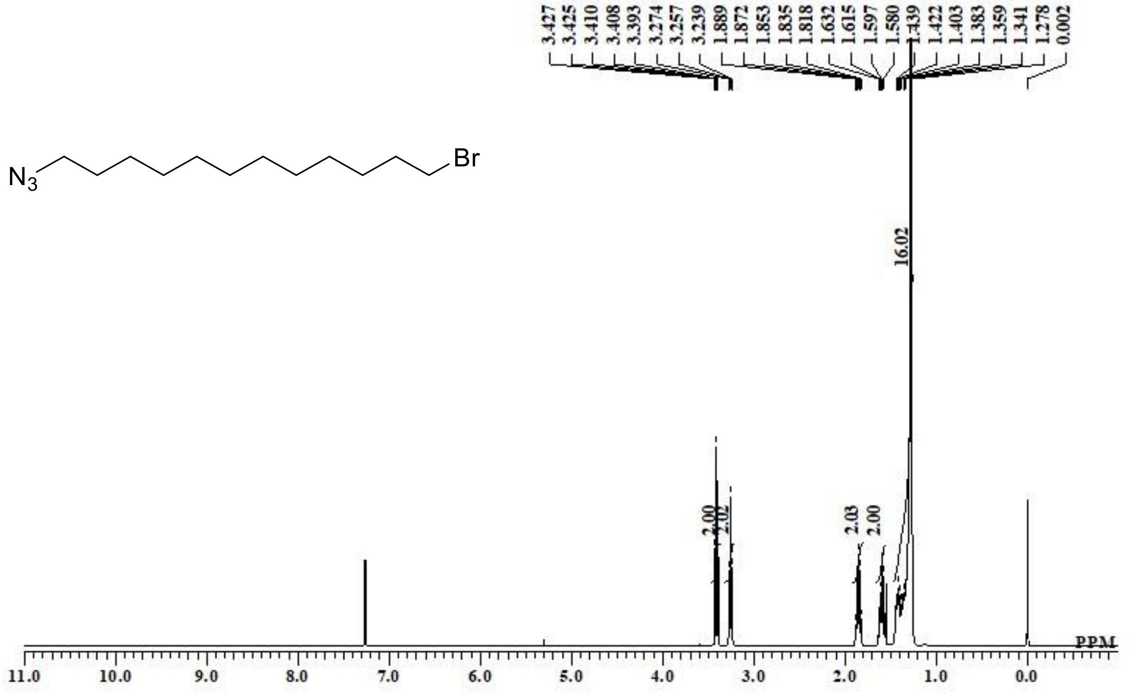

^{1}H-NMR: **18** ($CDCl_3$)

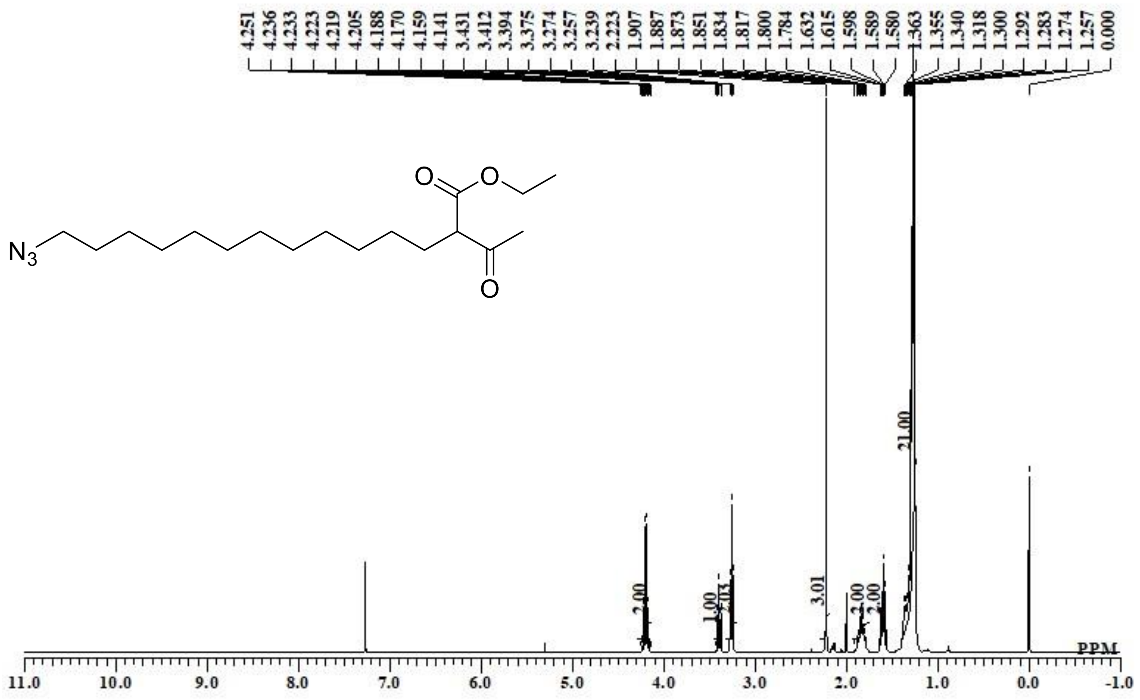

^{1}H-NMR: **19** ($CDCl_3$)

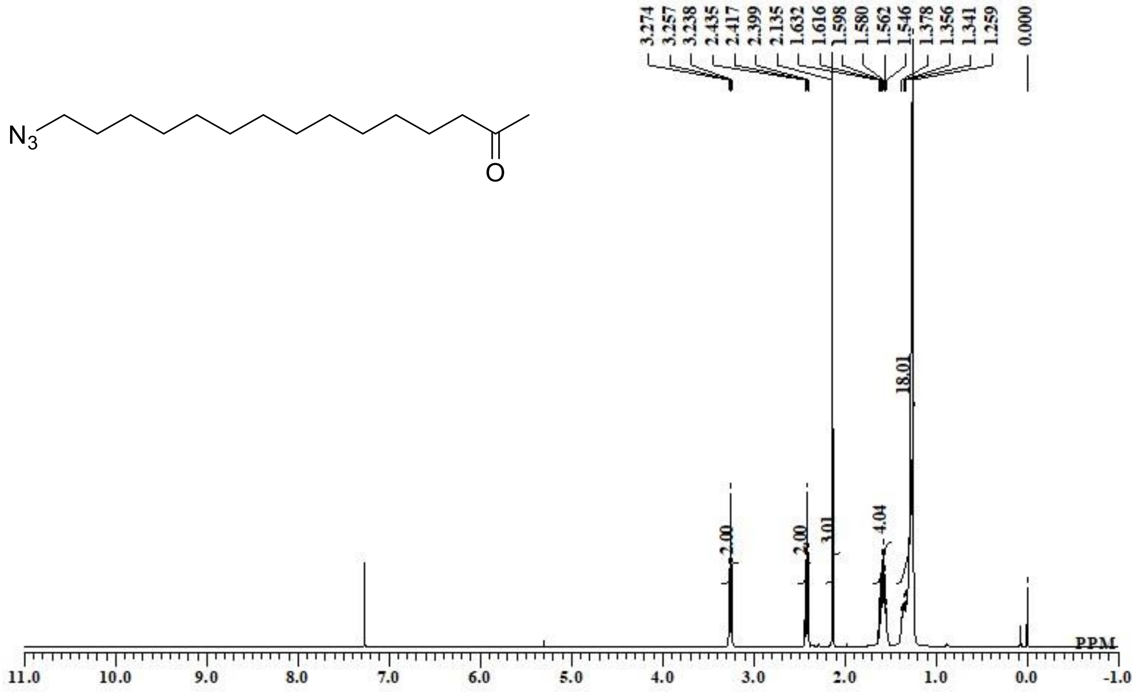

^{1}H-NMR: **20** ($CDCl_3$)

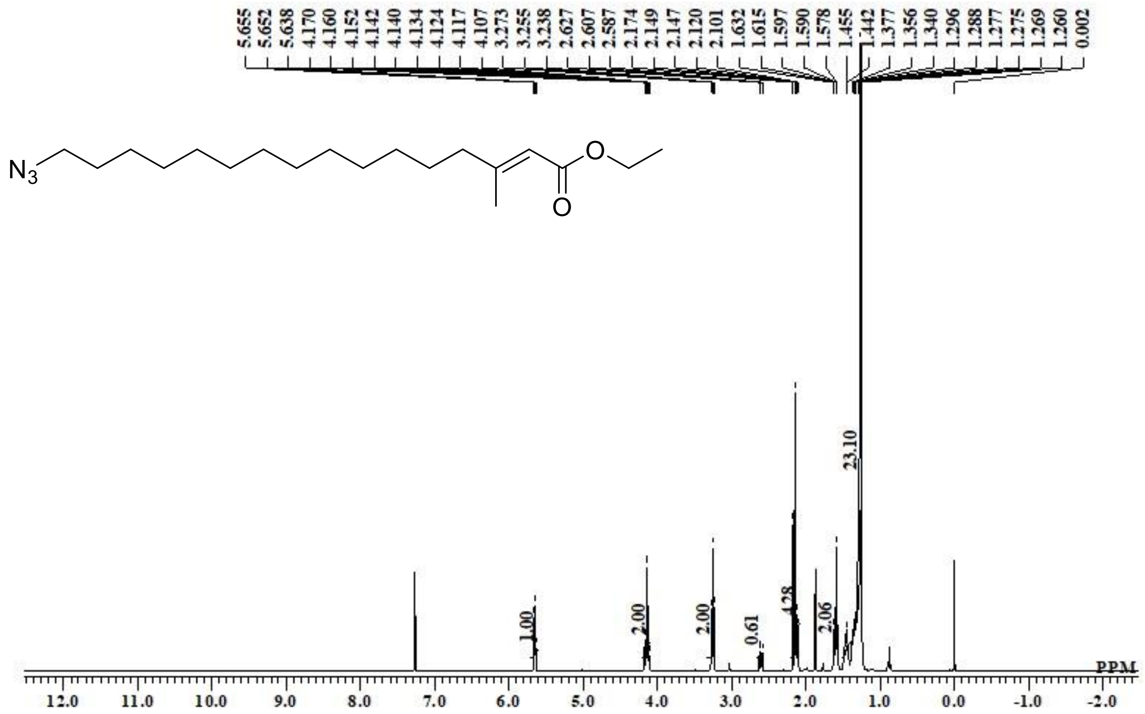

^{1}H-NMR: **21** ($CDCl_3$)

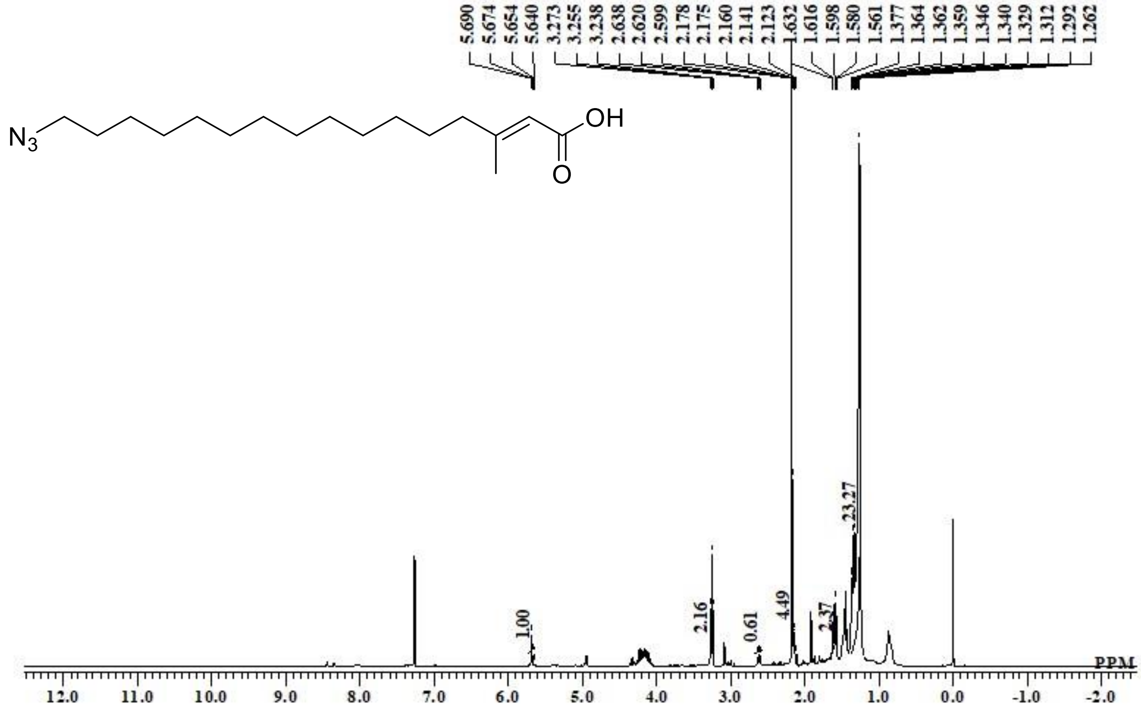

^{1}H-NMR: **22** (CD_3OD)

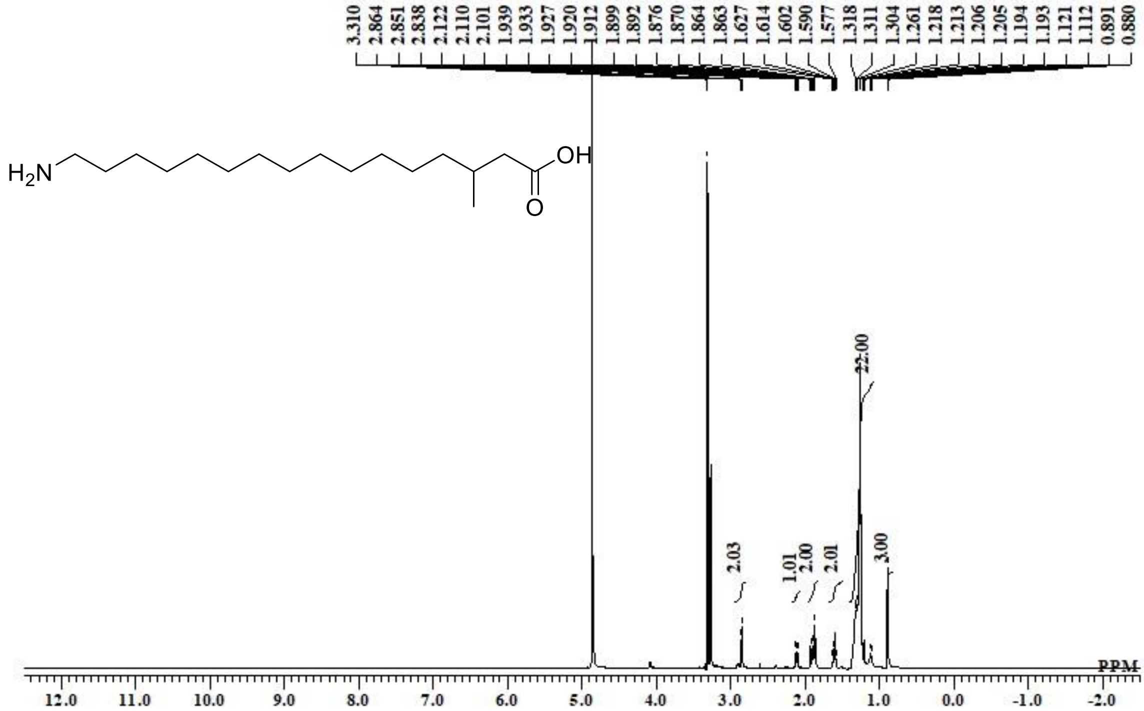

^{1}H-NMR: **24** ($CDCl_3$)

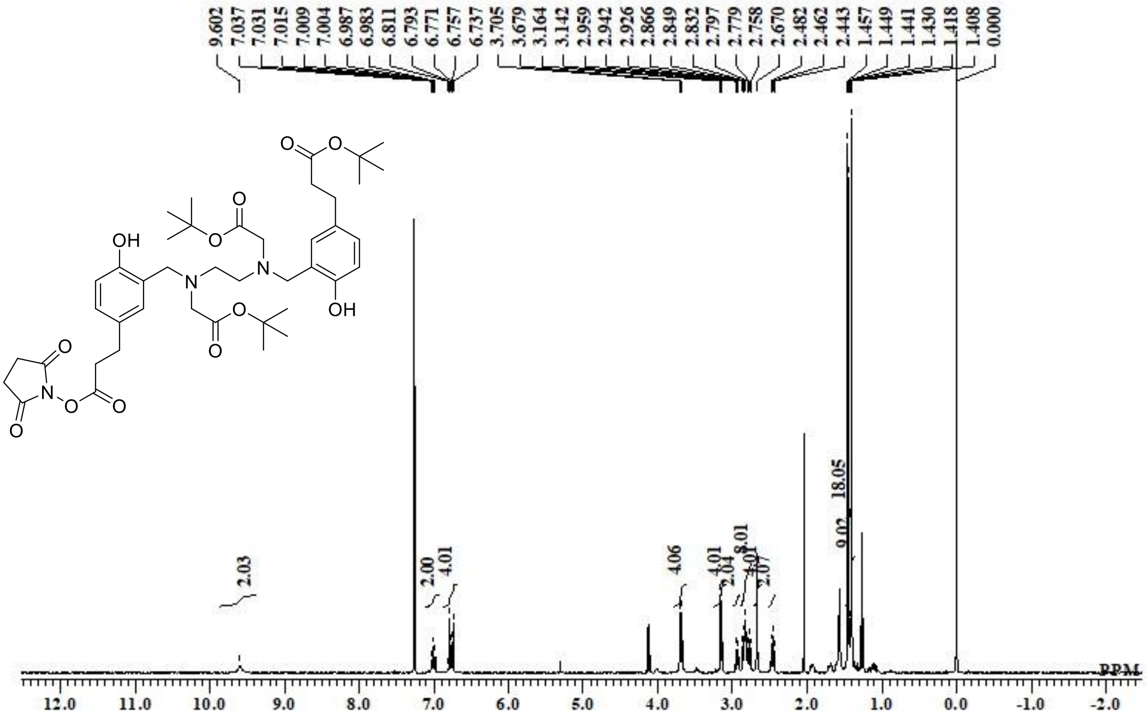

Supplement: S6 Fig — (PDF) [file pone.0261226.s006.pdf]
