# Supplementary material for: Synthesis and evaluation of radiogallium-labeled long-chain fatty acid derivatives as myocardial metabolic imaging agents
Source: PLoS One. 2021 Dec 15;16(12):e0261226. doi: 10.1371/journal.pone.0261226 (PMC8673672; doi:10.1371/journal.pone.0261226)
Supplement: S7 Fig — (PDF) [file pone.0261226.s007.pdf]

*HPLC chromatograms of metabolite analyses of [^{67}Ga]**5**, [^{67}Ga]**6**, [^{67}Ga]**7**, and [^{67}Ga]**8***

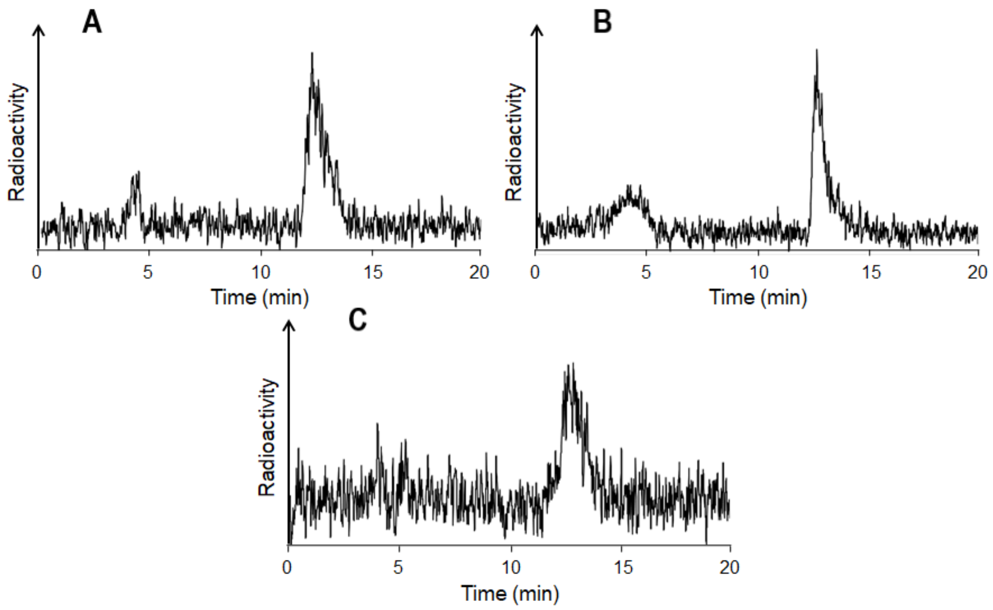

Fig S10A. Typical HPLC chromatograms of metabolite analyses from samples of blood (A), liver (B), and heart (C) in mice at 10 min postinjection of [^{67}Ga]**5**. HPLC system: Cosmosil 5C$_{18}$-AR-II column (4.6 mm ID × 150 mm) at a flow rate of 1.0 mL/min with a gradient mobile phase of 70–95% methanol in water with 0.1% TFA for 20 min, with UV detector at 254 nm wavelength, column temperature: 40 °C.

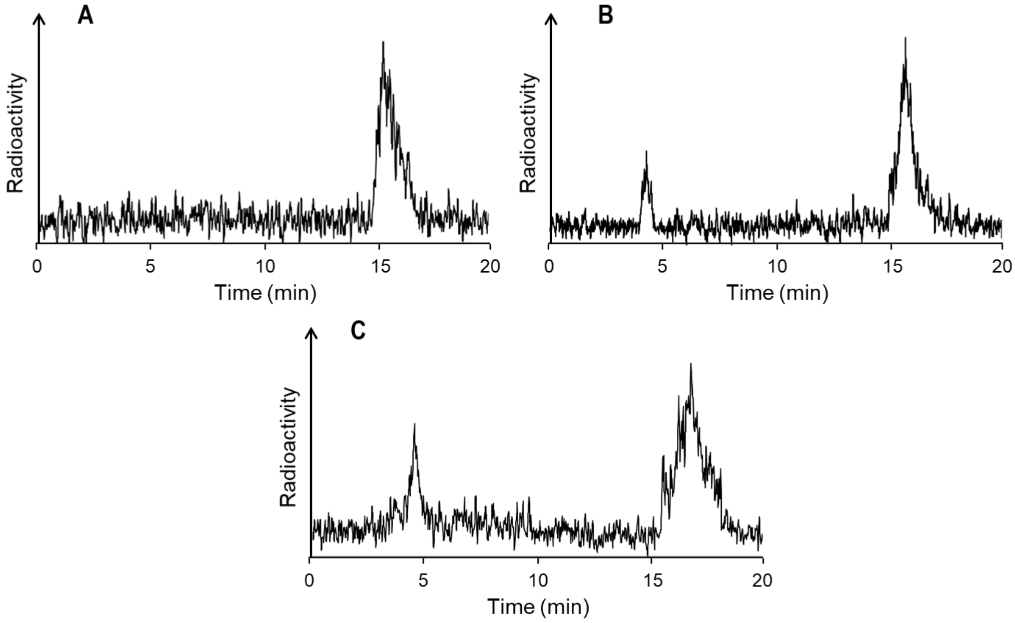

Fig S10B. Typical HPLC chromatograms of metabolite analyses from samples of blood (A), liver (B), and heart (C) in mice at 10 min postinjection of [^{67}Ga]**6**. HPLC system: Cosmosil 5C$_{18}$-AR-II column (4.6 mm ID × 150 mm) at a flow rate of 1.0 mL/min with a gradient mobile phase of 70–95% methanol in water with 0.1% TFA for 20 min, with UV detector at 254 nm wavelength, column temperature: 40 °C.

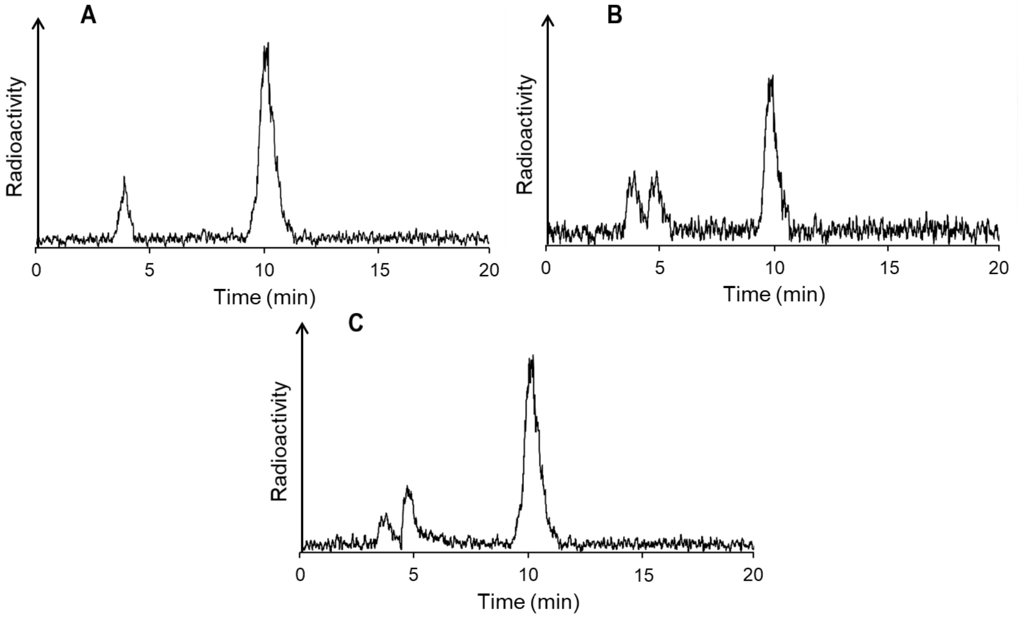

Fig S10C. Typical HPLC chromatograms of metabolite analyses from samples of blood (A), liver (B), and heart (C) in mice at 10 min postinjection of [^{67}Ga]**7**. HPLC system: Cosmosil 5C_{18}-AR-II column (4.6 mm ID × 150 mm) at a flow rate of 1.0 mL/min with a gradient mobile phase of 70–95% methanol in water with 0.1% TFA for 20 min, with UV detector at 254 nm wavelength, column temperature: 40 °C.

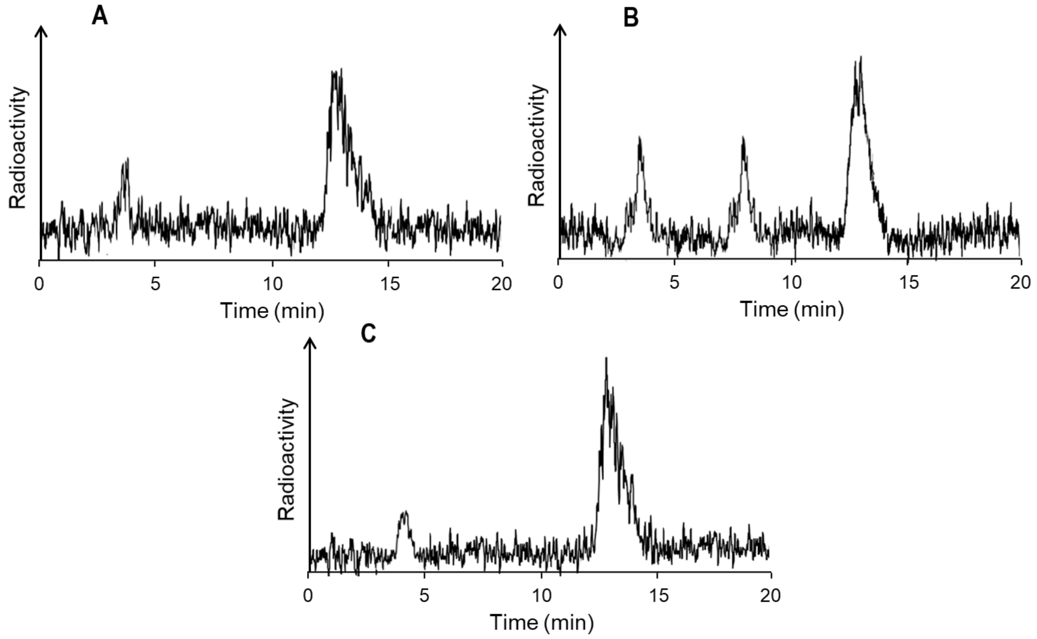

Fig S10D. Typical HPLC chromatograms of metabolite analyses from samples of blood (A), liver (B), and heart (C) in mice at 10 min postinjection of [^{67}Ga]**8**. HPLC system: Cosmosil 5C_{18}-AR-II column (4.6 mm ID × 150 mm) at a flow rate of 1.0 mL/min with a gradient mobile phase of 70–95% methanol in water with 0.1% TFA for 20 min, with UV detector at 254 nm wavelength, column temperature: 40 °C.
