# Supplementary material for: Synthesis and evaluation of radiogallium-labeled long-chain fatty acid derivatives as myocardial metabolic imaging agents
Source: PLoS One. 2021 Dec 15;16(12):e0261226. doi: 10.1371/journal.pone.0261226 (PMC8673672; doi:10.1371/journal.pone.0261226)
Supplement: S1 File — (DOCX) [file pone.0261226.s008.docx]

**Synthesis of 15-aminopentadecanoic acid (APDA)**

**Synthesis scheme of 15-aminopentadecanoic acid.** i) SOCl_2_, methanol, reflux, overnight; ii) MsCl, TEA, DCM, rt, 1 h; iii) NaN_3_, DMF:H_2_O (5:1), 80 °C, overnight; iv) 2.5 M NaOH, ethanol, H_2_O, rt, overnight; v) 10% Pd(OH)_2_/C, H_2_, methanol, rt, 5 h.

- 1. Methyl 15-hydroxypentadecanoate (**10**)

To an ice cooled dry methanol (25 mL) was added 15-hydroxypentadecanoic acid (**9**) (600 mg, 2.3 mmol), followed by thionyl chloride (SOCl_2_) (1.9 mL, 23.2 mmol) dropwise. After reflux for overnight, the reaction mixture was concentrated under reduced pressure. The residue was poured over saturated aqueous sodium hydrogen carbonate (NaHCO_3_) (35 mL), and extracted with dichloromethane (DCM) (3 × 40 mL). The combined organic layers were dried over magnesium sulfate (MgSO_4_) anhydrous, filtered, and concentrated under reduced pressure to obtain **10** (610 mg, 97%) as a colorless solid. ^1^H NMR (400 MHz, CDCl_3_): δ 3.67 – 3.64 (5H, m), 2.30 (2H, t, J = 7.2), 1.64 – 1.53 (4H, m), 1.34 – 1.23 (20H, m).

- 1. Methyl 15-[(methylsulfonyl)oxy]pentadecanoate (**11**)

To an ice cooled mixture of **10** (560 mg, 2.1 mmol) and triethylamine (TEA) (1.7 mL, 12.4 mmol, 6.0 eq.) in DCM (20 mL) was added methane sulfonyl chloride (MsCl) (0.5 mL, 6.2 mmol) dropwise. After stirring at room temperature for 1 h, the reaction was quenched with water. The reaction mixture was extracted with DCM (3 × 40 mL). After washing with hydrochloric acid (0.1 M HCl), water, and brine successively, combined organic layers were dried over MgSO_4_ anhydrous, filtered, and concentrated under reduced pressure to obtain **11** (710 mg, 98.5%) as a pale brown solid. ^1^H NMR (400 MHz, CDCl_3_): δ 4.22 (2H, t, *J* = 6.4 Hz), 3.67 (3H, s), 3.00 (3H, s), 2.30 (2H, t, *J* = 8.0 Hz), 1.78 – 1.71 (2H, m), 1.64 – 1.59 (2H, m), 1.40 – 1.26 (20H, m).

- 1. Methyl 15-azidopentadecanoate (**12**)

To a mixed solvent (dimethylformamidine (DMF) : H_2_O = 5 : 1) (10 mL) was added **11** (700 mg, 2.0 mmol) and sodium azide (NaN_3_) (1.5 g, 22 mmol). After stirring at 80 °C for overnight, water was added into the mixture to quench the reaction. The reaction mixture was extracted with a mixture of hexane and ethyl acetate (1/1) (3 × 25 mL). The combined organic layers were dried over MgSO_4_ anhydrous, filtered, and concentrated under reduced pressure to obtain **12** (550 mg, 92%) as a colorless oil. ^1^H NMR (400 MHz, CDCl_3_): δ 3.67 (3H, s), 3.26 (2H, t, *J* = 6.8 Hz), 2.31 (2H, t, *J* = 7.6 Hz), 1.64 – 1.58 (4H, m), 1.38 – 1.26 (20H, m).

- 1. 15-Azidopentadecanoic acid (**13**)

To a solution of **12** (580 mg, 1.95 mmol) in ethanol (5 mL), was added aqueous sodium hydroxide (2.5 M NaOH, 3 mL) dropwise. After being stirred at room temperature for overnight, the pH of reaction mixture was adjusted to 5.0 with 1 M HCl. The crude product was extracted with ethyl acetate (3 × 10 mL). The combined organic layers were dried over MgSO_4_ anhydrous, filtered, and concentrated under reduced pressure to obtain **13** (550 mg, 100%) as a colorless oil. ^1^H NMR (400 MHz, CDCl_3_): δ 3.26 (2H, t, *J* = 7.2 Hz), 2.35 (2H, t, *J* = 7.2 Hz), 1.67 – 1.56 (4H, m), 1.35 – 1.24 (20H, m).

- 1. 15-Aminopentadecanoic acid (APDA) (**14**)

To a solution of **13** (500 mg, 1.76 mmol) in dry methanol (10 mL) was added 10% Pd(OH)_2_/C (200 mg) and the mixture was stirred at room temperature for 5 h under hydrogen atmosphere. After removing the catalyst by filtration through a pad of Celite^®^, the filtrate was concentrated under reduced pressure to afford **14** (440 mg, 97%) as a colorless solid. ^1^H NMR (400 MHz, CD_3_OD): δ 2.43 (2H, t, *J ­*= 7.2), 2.21 – 2.18 (2H, m), 1.67 – 1.57 (4H, m), 1.44 – 1.30 (22H, m).
