# Supplementary material for: Synthesis and evaluation of radiogallium-labeled long-chain fatty acid derivatives as myocardial metabolic imaging agents
Source: PLoS One. 2021 Dec 15;16(12):e0261226. doi: 10.1371/journal.pone.0261226 (PMC8673672; doi:10.1371/journal.pone.0261226)
Supplement: S2 File — (DOCX) [file pone.0261226.s009.docx]

**Synthesis of 16-Amino-3-methylhexadecanoic acid (AMHDA)**

**Synthesis scheme of 16-amino-3-methylhexadecanoic acid.** i) 47% HBr, 95% H_2_SO_4_, reflux, overnight; ii) NaN_3_, DMSO, rt, overnight; iii) ethylacetoacetate, NaH, NaI, DMF, 45 °C, overnight; iv) 2.5 M NaOH, dioxane, ethanol, 50 °C, overnight; v) triethyl phosphonoacetate, NaH, DMF, rt, overnight; vi) 2.5 M LiOH, dioxane, 60 °C, overnight; vii) Pd(OH)_2_/C, H_2_, methanol, rt, overnight.

- 1. 1, 12-Dibromododecane (**16**)

A solution of dodecane-1,12-diol (**15**) (6.3 g, 31.5 mmol) in the mixture of 47% hydrobromic acid (HBr) (60 mL) and 95% sulfuric acid (H_2_SO_4_) (3 mL) was refluxed overnight. After cooling to room temperature, the mixture was diluted with water and extracted with hexane (3 × 100 mL). The combined organic layers were dried over MgSO_4_ anhydrous, filtered, and concentrated under reduced pressure. The crude product was purified by column chromatography on silica gel (hexane 100%) to obtain **16** (9.3 g, 90%) as a colorless solid. ^1^H NMR (400 MHz, CDCl_3_): δ 3.41 (4H, t, J = 6.8 Hz), 1.89 – 1.82 (4H, m), 1.44 – 1.39 (4H, m), 1.38 – 1.28 (12H, m).

- 1. 1-Azido-12-bromododecane (**17**)

To the solution of **16** (6.0 g, 18.3 mmol) in dimethyl sulfoxide (DMSO) (50 mL) was added NaN_3_ (1.2 g, 18.3 mmol). After stirring at room temperature for overnight, water (40 mL) was added to quench the reaction and followed by extraction with hexane (3 × 100 mL). The combined organic layers were dried over MgSO_4_ anhydrous, filtered, and concentrated under reduced pressure. The crude product was purified by column chromatography on silica gel (hexane 100%) to obtain **17** (2.4 g, 45.5%) as a colorless oil. ^1^H NMR (400 MHz, CDCl_3_): δ 3.41 (2H, t, *J* = 6.8 Hz), 3.26 (2H, t, *J* = 7.2 Hz), 1.89 – 1.82 (2H, m), 1.63 – 1.58 (2H, m), 1.44 – 1.28 (16H, m).

- 1. Ethyl 2-acetyl-14-azidotetradecanoate (**18**)

To an ice cooled suspension of sodium hydride (NaH) (60% dispersion in mineral oil, 400 mg, 10.3 mmol) in dry DMF (10 mL), ethylacetoacetate (1.3 mL, 10.3 mmol) was added and the mixture was stirred for 30 min. After adding **17** (1.0 g, 3.4 mmol) and sodium iodide (NaI) (500 mg, 3.4 mmol) the mixture was stirred at 45 °C overnight. Upon completion of the reaction, water was added to quench the reaction. The crude product was extracted with a mixture of hexane and ethyl acetate (1/1) (3 × 25 mL). The combined organic layers were dried over MgSO_4_ anhydrous, filtered, and concentrated under reduced pressure. The crude product was purified by column chromatography on silica gel (hexane/ethyl acetate = 15/1) to obtain **18** (860 mg, 74%) as a colorless oil. ^1^H NMR (400 MHz, CDCl_3_): δ 4.25 – 4.14 (2H, m), 3.39 (1H, t, *J* = 7.6 Hz), 3.26 (2H, t, *J* = 7.2 Hz), 2.22 (1H, s), 1.91 – 1.78 (2H, m), 1.63 – 1.58 (2H, m), 1.36 – 1.26 (21H, m).

- 1. 15-Azidopentadecan-2-one (**19**)

To a solution of **18** (860 mg, 2.5 mmol) in ethanol (3 mL), was added 2.5 M NaOH (5 mL) dropwise. After being stirred at 50 °C overnight, the pH of the reaction mixture was adjusted to 5.0 with 1 M HCl. The crude product was extracted with DCM (3 × 25 mL). The combined organic layers were dried over MgSO_4_ anhydrous, filtered, and concentrated under reduced pressure to obtain **19** (670 mg, 100%) as a colorless oil. ^1^H NMR (400 MHz, CDCl_3_): δ 3.26 (2H, t, *J* = 6.8 Hz), 2.42 (2H, t, *J* = 7.2 Hz), 2.14 (3H, s), 1.63 – 1.55 (4H, m), 1.38 – 1.26 (18H, m).

- 1. Ethyl (E)-16-azido-3-methylhexadec-2-enoate (**20**)

To an ice cooled solution of triethyl phosphonoacetate (1.4 gram, 6.3 mmol) in dry DMF (4 mL), NaH (60% dispersion in mineral oil, 253 mg, 6.3 mmol, 2.5 eq.) was added and the mixture was stirred for 1 h. After adding a solution of **19** (670 mg, 2.5 mmol) in dry DMF (1 mL), the mixture was stirred at room temperature for overnight. After completion of the reaction, aqueous saturated ammonium chloride (NH_4_Cl) was added to quench the reaction. The crude product was extracted with a mixture of hexane and ethyl acetate (1/1) (3 × 25 mL). The combined organic layers were dried over MgSO_4_ anhydrous, filtered, and concentrated under reduced pressure. The crude product was purified by column chromatography on silica gel using hexane (100%) to obtain **20** (660 mg, 78.4%) as a colorless oil. ^1^H NMR (400 MHz, CDCl_3_): δ 5.65 (1H, s), 4.17 – 4.11 (2H, m), 3.26 (2H, t, *J* = 7.2 Hz), 2.17 – 2.10 (5H, m), 1.63 – 1.58 (2H, m), 1.46 – 1.26 (23H, m).

- 1. (E)-16-Azido-3-methylhexadec-2-enoic acid (**21**)

To a solution of **20** (600 mg, 1.8 mmol) in ethanol (3 mL), was added 1,4-dioxane (3 mL) and aqueous lithium hydroxide (2.5 M LiOH, 6 mL) dropwise. After stirring at 60 °C overnight, the pH of the reaction mixture was adjusted to 5.0 with 1 M HCl. The crude product was extracted with diethyl ether (3 × 25 mL). The combined organic layers were dried over MgSO_4_ anhydrous, filtered, and concentrated under reduced pressure to obtain **21** (550 mg, 100%) as a colorless oil. ^1^H NMR (400 MHz, CDCl_3_): δ 5.69 (1H, s), 3.26 (2H, t, *J* = 7.2 Hz), 2.18 – 2.12 (5H, m), 1.63 – 1.56 (2H, m), 1.38 – 1.26 (20H, m).

- 1. 16-Amino-3-methylhexadecanoic acid (AMHDA) (**22**)

To a solution of **21** (500 mg, 1.62 mmol) in dry methanol (10 mL), was added 10% Pd(OH)_2_/C (200 mg) and the mixture was stirred at room temperature overnight under hydrogen atmosphere. After removing the catalyst by filtration through a pad of Celite^®^, the filtrate was concentrated under reduced pressure to afford **22** (330 mg, 71%) as a colorless solid. ^1^H NMR (400 MHz, CD_3_OD): δ 2.85 (2H, t, *J ­*= 5.2), 2.12 – 2.10 (1H, m), 1.94 – 1.86 (2H, m), 1.63 – 1.58 (2H, m), 1.31 – 1.11 (22H, m), 0.88 (3H, d, *J* = 4.4 Hz).
