# Supplementary material for: Synthesis and evaluation of radiogallium-labeled long-chain fatty acid derivatives as myocardial metabolic imaging agents
Source: PLoS One. 2021 Dec 15;16(12):e0261226. doi: 10.1371/journal.pone.0261226 (PMC8673672; doi:10.1371/journal.pone.0261226)
Supplement: S3 File — (DOCX) [file pone.0261226.s010.docx]

**Synthesis of HBED-CC(tBu)_3_-NHS ester**

**Synthesis scheme of HBED-CC(*t*Bu)_3_-NHS ester.** i) NHS, DCC, THF, rt, 5 h.

3-(3-{[(Carboxymethyl){2-[(carboxymethyl)(5-{3-[(2,5-dioxopyrrolidin-1-yl)oxy]-3-oxopropyl}-2-hydroxybenzyl)amino]ethyl}amino]methyl}-4-hydroxyphenyl)propanoic acid (HBED-CC(tBu)_3_-NHS ester) (**24**)

A mixture of HBED-CC-tris(tert-butyl) ester (**23**) (85 mg, 0.12 mmol), which was synthesized according to the previous report ^1^, N-hydroxysuccinimide (NHS) (17.3 mg, 0.15 mmol), and N, N'-dicyclohexylcarbodimide (DCC) (27.2 mg, 0.13 mmol) in dry THF (1 mL) was stirred at room temperature for 5 h. After removing the formed precipitate by filtration, the filtrate was concentrated under reduced pressure and the residue was purified by column chromatography on silica gel (hexane/ethyl acetate = 2/1) to obtain **24** (60 mg, 63%) as a colorless oil. ^1^H NMR (400 MHz, CDCl_3_): δ 9.60 (1H, s-br), 7.04 – 6.98 (2H, m), 6.81 – 6.74 (4H, m), 3.69 (4H, d, J = 10.4 Hz), 3.15 (4H, d, J = 8.8 Hz), 2.96 – 2.93 (2H, m), 2.87 – 2.76 (8H, m), 2.67 (4H, s), 2.46 (2H, t, J = 7.6 Hz), 1.45 (18H, d, J = 3.2 Hz), 1.41 (9H, s).

**Reference**

1 Makarem, A., Konrad, M., Liolios, C. & Kopka, K. A Convenient Synthesis for HBED-CC-tris(tert-butyl ester). *Synlett* **29**, 1239-1243, doi:10.1055/s-0036-1591950 (2018).
