# Supplementary material for: Synthesis and evaluation of radiogallium-labeled long-chain fatty acid derivatives as myocardial metabolic imaging agents
Source: PLoS One. 2021 Dec 15;16(12):e0261226. doi: 10.1371/journal.pone.0261226 (PMC8673672; doi:10.1371/journal.pone.0261226)
Supplement: Attachment — Submitted filename: Comments of reviewers_final.docx [file pone.0261226.s011.docx]

Reviewer#1: PONE-D-21-24937

We would like to thank the reviewer for the helpful comments. Please read the responses to your comments/suggestions below:

1. The scheme shown for the synthesis of synthesis of 16-Amino-3-methylhexadecanoic acid (AMHDA) is incorrect. The conversion of Dibromo derivative to azide and OH groups is not possible.

**Answer:** As the reviewer suggested, the structure in the scheme had been incorrect. We corrected the structure in the scheme. The synthetic procedure and ^1^H NMR data are correct and do not need to be changed.

2. The mass spectra are missing.

**Answer**: We tried to analyze the molecular weight of **17** using ESI-MS. However, it was difficult. The possible reason could be that this intermediate compound is poorly ionized; therefore, we just showed H-NMR data.

3. The 1H NMR suggest that the compounds are not pure. 13C NMR would provide clear picture, hence are required. The final application of the developed systems is in-vivo, so high purity of the systems is a necessity.

**Answer**: ^1^H NMR spectra of these compounds contained broad signals derived from a cyclene ring under equilibrium of some conformers. We tried two different solvents (DMSO *d*_6_ and methanol *d*_4_), however, the broadening was not solved. ^13^C NMR also showed broad signal and was difficult to assign. Nevertheless, We think purity of HBED-CC-PDA (**1**), HBED-CC-MHDA (**2**), DOTA-PDA (**3**), and DOTA-MHDA (**4**) are high because all of non-broad signals of these spectra are derived from these compounds. Moreover, we determined the high purities of **1**-**4** by HPLC. We showed their chromatograms in Fig S4.

4. The most of the compounds prepared are in mg. Was the quantity sufficient enough to perform the characterization and other studies?

**Answer**: 2-3 mg of precursors for the characterization using H-NMR and MS and 100 µg of nonradioactive Ga-complex for the characterization using MS are enough. For other studies using radiolabeled compounds, 20 µg of precursors are needed for radiolabeling. Thus, the quantity we synthesized is sufficient to perform the characterization and other studies.

5. What was the specific activity of the Ga derivatives.

**Answer**:

As the reviewer suggested, we added following sentences in Page 19 – 20 line 380 - 388.

“The radiolabeling was performed with carrier-free radionuclides and under non-carrier-added conditions. [^67^Ga]**5** and [^67^Ga]**6** were entirely separated from the corresponding precursors, **1** and **2**, by the HPLC purification. Thus, the molar activity of [^67^Ga]**5** and [^67^Ga]**6** was estimated to be approximately 1.5 × 10^18^ Bq/mol. Meanwhile, the molar activity of [^67^Ga]**7** and [^67^Ga]**8** was determined to be 1.1 × 10^15^ Bq/mol and 1.2 × 10^15^ Bq/mol, respectively. These values were much lower than that of [^67^Ga]**5** and [^67^Ga]**6** because [^67^Ga]**7** and [^67^Ga]**8** were not entirely separated from the corresponding precursors.”

6. The stability of the Ga derivatives are in the range of 85% post 1h, which is poor. The metabolite studies are necessary to negate the false positive results.

**Answer**: We performed metabolite studies of these radiolabeled compounds in blood, liver, and heart. We attached the data in the revised manuscript. A protocol of the experiment is described at Page 17 – 18 line 337 – 346 and results and discussion of the metabolite study are described at Page 29 line 531 – 549.

7. The developed compound even post methylation did not display high uptake in heart thus proves the hypothesis wrong.

**Answer**: As the reviewer pointed out, the results were out of our expectations. We expected that the methylation could increase the accumulation of radioactivity in the heart because it is known that methyl-branched fatty acids can prolong cardiac retention. However, the methylation did not affect it. First of all, higher initial uptake in the heart must be necessary. We had mentioned them in our manuscript as follows.

Page 27 line 504 – 509 (result and discussion) “An introduction of a *β*-methyl group in fatty acid analogs, such as [^123^I]BMIPP, has been known to delay the washout from the heart due to interference with its *β*-oxidation (37). Thus, we synthesized radiogallium-labeled fatty acid analogs with and without *β*-methyl group and compared their biodistribution. However, tracer retention in the heart was not greatly changed against our expectation. This may be caused by their low initial uptakes.”

Page 30 line 555 – 559 (conclusion) “Contrary to our expectation, they cannot be applied as PET myocardial metabolic imaging agents due to their low accumulation in the heart. Therefore, structural modifications, especially in the chelate site, are required to develop ^68^Ga-labeled fatty acids with adequate accumulation in the heart as myocardial metabolic imaging agents.”

8. Also the uptake of the four derivatives in the heart is far less than 123I- BMIPP. Comparative studies with the other known systems would be useful to understand the efficacy of the systems developed.

**Answer**: We added the comparison between radiogallium labeled fatty acids evaluated in this study and those evaluated in previous studies in the results and discussion section.

Reviewer #2: excellent work and good effort to introduce the new chemical scaffold for exploring this area. this new class of the analogs will further explores to enhance the knowledge of these kind of radio pharmaceuticals

**Answer**: Thank you for your appreciation of our study.
